# Effects of Fermentation Period on Metabolite Production and Bioactivity in Two Sponge‐Associated Bacteria

**DOI:** 10.1155/sci5/5979734

**Published:** 2026-07-29

**Authors:** Mamdouh Al-Harbi, Mamdoh T. Jamal, Sathianeson Satheesh

**Affiliations:** ^1^ Department of Marine Biology, Faculty of Marine Sciences, King Abdulaziz University, Jeddah, Saudi Arabia, kau.edu.sa

**Keywords:** antibiofilm, bioactive compounds, natural products, Red Sea, sponge-associated bacteria

## Abstract

Many marine bacteria are living in association with sponges and are known to produce secondary metabolites for the host’s chemical defense. The secondary metabolite production in bacteria is influenced by the culture medium, growth conditions, and incubation time. In this study, the effect of fermentation period on secondary metabolite production was analyzed in two sponge‐associated bacteria, *Enterobacter* sp. and *Alcanivorax* sp. The bacterial strains were subjected to a fermentation period of five days and then a 14‐day period before centrifugation and solvent extraction from the supernatant. The extracts from the bacteria were then tested for antibacterial and antibiofilm activity against selected clinically significant bacterial strains. The composition of the extracts was confirmed using GC–MS. The results revealed variations in the activity of the extracts at different fermentation periods with differences in the composition of the metabolites observed in each extract. *Enterobacter* sp. cultured for 14 days of fermentation period showed higher antibacterial activity against *Staphylococcus aureus* ATCC 25923 and *Escherichia coli* ATCC 25922. While each extract demonstrated antibiofilm activity, in some cases the activity was higher after 5 days and in some cases after 14 days. GC–MS profiling clearly indicated a metabolic shift with the dominance of fatty alcohols and esters in the extracts obtained after 5 days and bioactive aromatic compounds and diketopiperazines after 14 days of fermentation. Overall, this study confirms that secondary metabolite enrichment in marine bacteria is fermentation time‐dependent, and a longer fermentation period may enhance antibacterial activity. The changes in metabolites due to the fermentation period showed the significance of optimizing bacterial culture time to enhance the bioactive potentials of bacteria associated with marine organisms.

## 1. Introduction

Sponges are among the most remarkable benthic marine invertebrate communities that cope with ecological threats and toxic compounds in water [1]. Sponges rank among the most resilient benthic marine invertebrates, thriving amid ecological threats and toxins through symbiotic associations with diverse microbes [2]. These microorganisms drive nutrient cycling and produce secondary metabolites for sponge chemical defense [3], positioning them as key sources of novel bioactive compounds [4]. This role in sponge chemical defense makes the microbes important sources of novel compounds [5, 6]. The association provides a source of natural compounds with potential application as novel pharmaceutical agents [7, 8]. New sources of pharmaceutical compounds are required to combat the challenge of antimicrobial resistance, which is responsible for an estimated 1.3 million annual deaths, making it deadlier than HIV/AIDS and malaria combined [9].

Culturable sponge‐associated bacteria, including *Enterobacter* and *Alcanivorax* spp., are potent producers of metabolites like diketopiperazines (cyclic dipeptides with antimicrobial/antibiofilm properties) and fatty acid derivatives [10]. *Enterobacter* species isolated from marine sponges have been shown to produce polyketides and peptides under optimal conditions [11], while *Alcanivorax* species can biosynthesize antibiofilm agents like long‐chain alcohols [12]. However, previous studies have shown inconsistencies: short fermentations (e.g., 24–72 h) tend to favor the production of fatty esters in *Alcanivorax* [13–15], whereas extended periods (7–14 days) tend to result in the enrichment of diketopiperazines and alkanes in *Enterobacter*‐like strains, boosting activity against *S. aureus* and *E. coli* [16–19]. Critically, these studies often fail to consider strain‐specific optimal conditions; for instance, nutrient stress in prolonged cultures can trigger diketopiperazine accumulation via quorum sensing [20], but this approach carries the risks of toxicity or dilution of the desired profile [21–23].

To ensure the production of effective metabolites and increase the maximum yield, there is a need to optimize the production condition of the bacterial strain and consider the factors that play a role in the growth of the metabolite‐producing bacteria. The selection of an appropriate growth medium and growth conditions is crucial in determining the bioactive compounds produced by sponge‐associated bacteria [24]. Screening of growth conditions is essential to produce a specific metabolite with a specific function [25].

Optimization of media, temperature, and incubation time is essential for maximizing yields [26], as bacteria adapt to harsh conditions to unleash defenses [27]. Fermentation duration profoundly shapes outputs for sponge‐associated bacteria since an organism produces different fermentation products during varying incubation periods [28, 29]. Previous studies have primarily focused on a single fermentation period or individual bioactivity studies using sponge‐associated bacteria [30–32]. A comparative study on the influence of different incubation periods on metabolite production and antibiofilm activity remains limited. Therefore, this study analyzes differences in metabolite production in Red Sea sponge (*Siphonochalina siphonella*)‐derived *Enterobacter* sp. (OM966411) and *Alcanivorax* sp. (ON514280) at 5‐ and 14‐day fermentations. The main objective was to understand the optimal incubation period for extracting bioactive metabolites from marine bacteria associated with sponges for antibacterial/antibiofilm extracts, addressing previous limitations through gas chromatography–mass spectrometry (GC–MS) profiling. It was hypothesized that increasing the fermentation period from 5 to 14 days would result in the accumulation of more bioactive metabolites due to stressful conditions leading to enhanced bioactivity.

## 2. Materials and Methods

### 2.1. Bacterial Strain

Sponge‐associated bacteria: *Enterobacter* sp. (OM966411) and *Alcanivorax* sp. (ON514280) were used in this study. These bacteria were isolated from a Red Sea sponge, *S. siphonella* [12, 29]. They were used in this study as a source of antibacterial and antibiofilm compounds following a solvent extraction process. The target bacterial isolates used in this study for antibacterial and antibiofilm assays were *Staphylococcus aureus* ATCC 25923 and *Escherichia coli* ATCC 25922. Others are *Acinetobacter baumannii*, a multidrug‐resistant bacterium, methicillin‐resistant *S. aureus*, and *S. aureus*, which were obtained from King Abdulaziz University Hospital (KAUH).

### 2.2. Culture Media and Chemicals

Culture media used in this study include the following: Zobell marine agar (from HiMedia, India) for the growth of *Enterobacter* sp. and *Alcanivorax* sp. and Zobell marine broth (ZMB) (HiMedia, India) were used as fermentation media for the metabolite extraction process. Also, Mueller–Hinton agar (Micromaster, India) for sensitivity tests and nutrient agar (Himedia, India) and blood agar for the culture, maintenance, and growth of target bacteria were used.

The chemicals used in this study include isopropanol (≥ 99%, Sigma‐Aldrich, Germany), methanol (≥ 99.8%, Sigma‐Aldrich, Germany), ethanol (≥ 99.8%, Honeywell, USA), and ethyl acetate (Honeywell, USA).

### 2.3. Optimization of the Medium and Cultivation Conditions

The two strains of sponge‐associated bacteria, *Enterobacter* sp. and *Alcanivorax* sp., were subjected to the solvent extraction method. The growth of the two bacterial isolates was optimized for temperature and incubation time to reach exponential phase while maintaining the pH of the medium (ZMB) at 7.7. The incubation temperature from 20°C to 35°C was adopted to select the optimum temperature for growth. Optical density at 600 nm (OD_600_) was measured with a spectrophotometer to monitor the growth as described previously [20]. The optimum temperature, which has the highest OD record for both strains, is 30°C. This temperature was maintained throughout the experiment. The method of Ortega‐Morales et al. [33] was adopted to estimate the incubation time required for each organism to reach the exponential phase. The organisms were incubated, and observation was made by measuring the OD_600_ after every hour. The exponential phase was attained after periods of 16 and 18 h, respectively, for *Enterobacter* sp. and *Alcanivorax* sp.

### 2.4. Extraction From Culture Supernatant

The organisms were inoculated in tubes containing 10 mL of ZMB and incubated overnight at 30°C until the exponential phase was attained. To extract secondary metabolites from the two strains, the overnight culture was transferred to a 1000 mL flask containing 500 mL ZMB. The flask was incubated at 30°C for 5 days and for 14 days using the same procedure as before with shaking at 180 rpm. The 5‐day fermentation period was selected to represent the metabolite production during the stationary phase based on previous studies that reported 5–7 days [24, 34]. The 14 days of fermentation represent the extended stationary phase combined with reduced nutrient conditions and stress. Culture supernatant was obtained by centrifugation at 8000 × g for 20 min. The supernatants obtained were then filtered to remove all remnants of bacterial cells. To confirm that the supernatant was a cell‐free supernatant (CFS), 100 μL was deposited on ZMA plates and cultured for 24 h at 30°C to observe if there was any growth. Absence of growth indicates that the supernatant was a CFS [35]. An equal volume of ethyl acetate was added to the CFS in flasks and agitated for 4 h in a shaker and separated in a separating funnel. The solvent fraction was filtered using Whatman No. 1 filter paper and then evaporated using vacuum evaporation. Crude extracts obtained were solubilized in dimethyl sulfoxide (DMSO) and adjusted to a concentration of 25 mg·mL^−1^. They were labeled as E5 and E14 for extracts from *Enterobacter* sp. extracted after 5 and 14 days, respectively, while A5 and A14 were applied to extracts from *Alcanivorax* sp.

### 2.5. Antibacterial Activity of the Extracts

The crude extracts were evaluated for antibacterial activity against five target bacteria using the well diffusion method. The bacteria are *S. aureus* ATCC 25923, *E. coli* ATCC 25922, *A. baumannii*, methicillin‐resistant *S. aureus* and *S. aureus*. Colonies from each bacterium in nutrient broth were incubated overnight at 37°C. The culture was adjusted to 0.5 McFarland standards at OD_600_. The surface of Mueller–Hinton agar plates was uniformly spread with the culture with a sterile cotton swab. Wells were perforated with a sterile cock borer of 6 mm diameter. The well was filled with 50 μL of the extracts or 100% DMSO and erythromycin as a control, followed by incubation at 37°C for 24 h. The experiment was conducted in replicates (*n* = 3). The activity of each extract was examined from the clear zone around the well and measured as the diameter of the zone of inhibition (ZI). The result was interpreted as strong when ZI ≥ 20 mm, moderate (ZI = 14–19 mm), weak (ZI ≤ 13 mm), or resistant when ZI = 0.

### 2.6. Biofilm‐Forming Ability of Target Bacterial Strains

All five organisms were evaluated for their ability to form biofilm on surfaces using the crystal violet (CV) assay in a 96‐well microtiter plate as described previously [36]. An inoculum for each organism was prepared by inoculating a colony into a tube containing nutrient broth and incubated overnight at 37°C. The inoculum was diluted in fresh nutrient broth and adjusted to a value of 0.2 at OD_600_. An aliquot of 100 μl of the inoculum was inoculated in the wells of a microtiter plate containing 100 μl of nutrient broth in triplicates and incubated statically at 37°C. After incubation, the unattached cells were removed by pouring out the content of the wells and then washed gradually with phosphate‐buffered saline (PBS) and allowed to dry before CV application. The CV was washed thrice with PBS and again allowed to dry [37]. Biofilm formation was observed directly for the cells that retain the CV stain. The confirmed biofilm bacteria selected are *S. aureus*, *A. baumannii*, and *S. aureus* ATCC 25923.

### 2.7. Antibiofilm Activity

The biofilm bacteria selected (*S. aureus*, *A. baumannii*, and *S. aureus* ATCC 25923) were first grown in NB overnight with shaking at 37 °C. The culture of each biofilm organism was diluted in fresh media (1:100). To the well of a 96‐well round‐bottom microplate (SPL Diagnostics), 100 μL of the culture was taken, and 100 μL of the extract was added. The control wells were maintained with 100 μL of bacterial culture and 100 μL of medium. The treated and untreated culture wells were maintained in replicates (*n* = 3). This was followed by incubation for 24 h at 37 °C. The unattached bacterial cells (planktonic growth form) were transferred to new 96‐well microtiter plates. Optical reading was taken of the new plate and was recorded with a microplate reader (Synergy, Biotek, USA) at 570 nm (OD_570_). The unattached bacterial cells that remained in the main plate were then washed thrice with PBS and allowed to dry on a damp paper. The wells were stained with 0.2% CV and stood for 20 min at room temperature. The CV stain was washed thrice with PBS. After drying, the wells were de‐stained with 95% ethanol using a multichannel pipette and shaken for 15 min at room temperature and then transferred to fresh 96‐well plates for recording the OD_570_ with a microplate reader. The planktonic growth inhibition and the biofilm formation inhibition activity were calculated from equations (1) and (2), respectively.

(1) Planktonic growth inhibition activity=OD control−OD sampleOD control×100,

where

OD sample = sample (culture and extract) optical density at 570 nm wavelength.

OD control = control (culture only) optical density at 570 nm wavelength.

(2) Percentage biofilm inhibition activity %=100−B×100C,

where

*B* = OD_570_ of biofilm culture in the presence of the extract.

*C* = OD_570_ of biofilm control without the addition of extract.

### 2.8. GC–MS Chemical Profile of the Extracts

GC–MS was used to analyze the chemical profile of the extracts using the GCMS‐QP2010 system (Shimadzu, Japan). Before injection of the sample, it was dissolved in isopropanol and filtered with a syringe filter. The column was programmed, and the analysis was carried out to identify the chemical compounds as described previously [38]. The analysis was conducted in replicates (technical replicates, *n* = 3) in each sample to confirm the results. The peaks of the compounds at different mass‐to‐charge ratios were the basis for tentative identification using the mass spectra library search of the National Institute of Standards and Technology using the similarity index or match factor (< 92%). The metabolite comparison was carried out based on peak area abundance. Database searches for the compounds with antimicrobial and antibiofilm properties were carried out in the biofilm database [39] and other literature.

### 2.9. Statistical Analysis

The data obtained from the biofilm inhibition assay were tested using one‐way ANOVA (*p* < 0.05 = significant) to understand the variations in antibiofilm activity between the two incubation period extracts. The analysis was carried out using the Statistica (Ver. 13) program. The GC–MS peak abundance of major tentatively identified compounds was used for heatmap analysis using SPSS (Ver. 27). Also, principal component analysis was conducted (Python, Ver. 3.13.5) on the peak abundance data of compounds tentatively detected in GC–MS analysis to understand the variation between the fermentation periods.

## 3. Results

### 3.1. Antibacterial Activity of the Extracts

The antibacterial activity against the growth of the tested organisms was revealed by the clear zone around the well, measured as the diameter of the ZI. All the extracts show various forms of activity, from weak to moderate and, in a rare case, strong activity. The results as presented in Table 1 show that extracts obtained after a 14‐day fermentation period have activity against five of the tested organisms. Strong activity was observed only by extract E14 against *S. aureus*. Extracts obtained from *Enterobacter* sp. and *Alcanivorax* sp. after 5 days have no activity against *S. aureus* ATCC 25923 and *S. aureus*, respectively. The most susceptible organisms are *A. baumannii* and MRSA that are sensitive to all four extracts.

**TABLE 1:** Measured zone of inhibition (mm) recorded by extracts obtained at different fermentation periods from sponge‐associated bacteria.

Extract	*Staphylococcus aureus*	Methicillin‐resistant *Staphylococcus aureus*	*Staphylococcus aureus* ATCC 25923	*Acinetobacter baumannii*	*Escherichia coli*
E5	15 ± 2	18 ± 1	0	9 ± 1	0
E14	20 ± 1.7	17 ± 1	15 ± 2	9 ± 0	9 ± 1
A5	0	12 ± 2	13 ± 1.7	12 ± 1	0
A14	9 ± 1	17 ± 2	13 ± 1	13 ± 1	0
Control (erythromycin)	23 ± 2	14 ± 1.7	31 ± 2	40 ± 3	14 ± 2

*Note:* E5 and E14 represent *Enterobacter* sp. after 5 and 14 days, respectively. A5 and A14 represent *Alcanivorax* sp. after 5 and 14 days, respectively. Values are presented as mean ± SD (*n* = 3).

### 3.2. Biofilm Inhibition Activity

**FIGURE 1:**
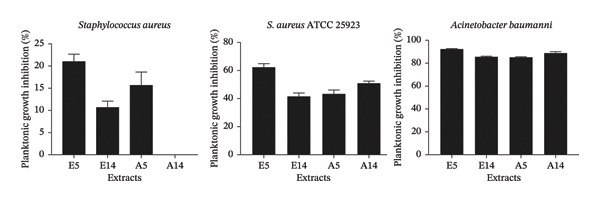
Planktonic growth inhibition activity of extracts from *Enterobacter* sp. (E5 and E14) and *Alcanivorax* sp. (A5 and A14) against some biofilm‐forming bacteria. E5 and A5 represent extracts obtained after a 5‐day fermentation period; E14 and A14 represent the extract after 14 days of fermentation. Values are expressed in mean ± SE. Error bars represent SE (*n* = 3).

**FIGURE 2:**
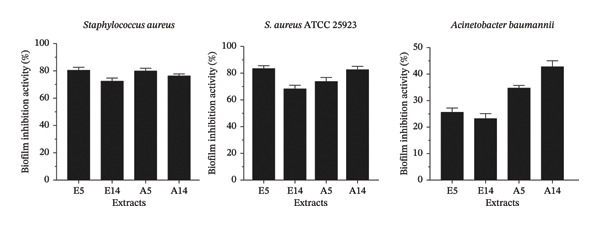
Antibiofilm activity of extracts from *Enterobacter* sp. (E5 and E14) and *Alcanivorax* sp. (A5 and A14) against some clinical biofilm strains. E5 and A5 represent extracts obtained after a 5‐day fermentation period; E14 and A14 represent the extract after 14 days of fermentation. Values are expressed in mean ± SE. Error bars represent SE (*n* = 3).

The antibiofilm activity was measured from the ability of the extracts to inhibit the planktonic stage of the biofilm process and the attachment stage. Inhibition as high as 93% was recorded by the extracts against *A. baumannii*, while *S. aureus* has the least inhibition, with zero activity recorded by extract A14 as indicated in (Figure 1). Biofilm inhibition activity was higher against the two *Staphylococcus* isolates, with *Acinetobacter* recording the least activity (Figure 2). Acinetobacter is a Gram‐negative (G−ve) organism, while the other two are Gram‐positive (G+ve) bacteria. The inhibition activity ranges from as low as 21% to as high as 84%. Extracts obtained after 5 days (A5 and E5) have higher activity against *S. aureus* than their counterparts. While the difference in biofilm inhibition between E5 and E14 against *S. aureus* was significant (*p* < 0.05), the extracts A5 and A14 did not show significant variation. E5 also has higher activity than E14 against *S. aureus* (*p* < 0.05) and *A. baumannii* not significant (*p* > 0.05), but the reverse is the case between A5 and A14, where A14 has higher inhibition activity (*p* < 0.05) than A5 against the said organisms.

### 3.3. Chemical Compounds Identified From the Extracts

**FIGURE 3:**
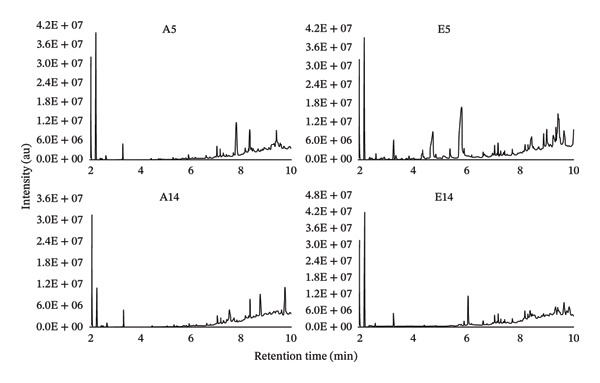
GC–MS spectra from the extracts of *Enterobacter* sp. (E5 and E14) and *Alcanivorax* sp. (A5 and A14). E5 and A5 represent extracts obtained after a 5‐day fermentation period; E14 and A14 represent the extract after 14 days of fermentation.

**FIGURE 4:**
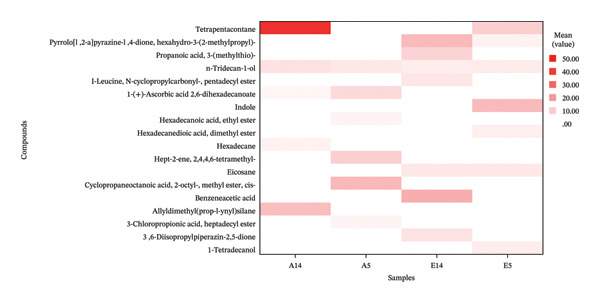
Heatmap of tentatively annotated compounds in the extracts of two bacterial strains obtained after 5 and 14 days of fermentation. E5 and E14 represent *Enterobacter* sp. after 5 and 14 days, respectively, A5 and A14 represent *Alcanivorax* sp. after 5 and 14 days, respectively. Heatmap analysis was conducted based on the peak abundance of each tentatively annotated metabolite in the GC–MS analysis.

**FIGURE 5:**
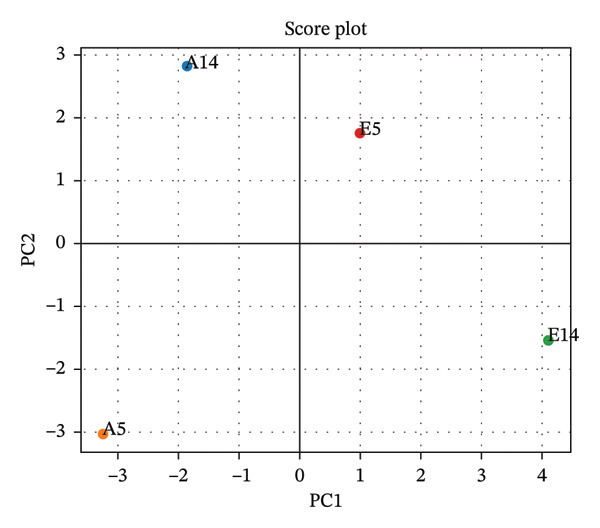
Principal component analysis of GC–MS metabolite profiles of extracts of two bacterial strains associated with a marine sponge. E5 and E14 represent *Enterobacter* sp. after 5 and 14 days, respectively, A5 and A14 represent *Alcanivorax* sp. after 5 and 14 days, respectively.

The fermentation period considerably influenced the chemical composition of the extracts obtained from two bacterial strains, as evidenced by GC–MS analysis. The GC–MS spectra of extracts obtained from the bacteria after the two incubation periods are shown in (Figure 3). The GC–MS chromatograms showed variations in peak number and intensity between the extracts, indicating the differences in their chemical composition due to fermentation periods. The heatmap analysis of metabolite distribution among the two different fermentation periods is shown in (Figure 4). The results from the GC–MS chemical profile analysis (tentative annotation of compounds) of the extracts from *Enterobacter* sp. and *Alcanivorax* sp. also revealed the impact of fermentation period on the extracts’ chemical composition (Supporting Tables S1–S4). Based on the number of different compounds tentatively identified, extracts E5 and E14 contained 7 compounds, followed by A5 with 6 compounds, and 5 compounds were detected in A14 (Supporting Tables S1–S4). The tentative identification showed that fatty acid esters were dominant in the extract obtained after 5 days, while diketopiperazines and long‐chain alkanes were enriched after 14 days of fermentation (Figure 4). Principal component analysis of the GC–MS tentatively annotated metabolite profile also revealed variations among the extracts, with PC1 and PC2 explaining 45.77% and 31.69%, respectively, of the total variance (Figure 5). The PCA score plot showed a difference in clustering of extracts between both fermentation periods in *Enterobacter* sp. and *Alcanivorax* sp., indicating variations in the chemical composition of the extracts.

## 4. Discussion

Sponge‐associated bacteria produce secondary metabolites that protect their hosts, with many showing therapeutic potential as bioactive compounds [38]. This study investigated how fermentation period affects metabolite production and activity in two sponge‐associated bacteria, *Enterobacter* sp. and *Alcanivorax* sp. The growth conditions were optimized and fermented the bacteria for 5 days (normal period) or 14 days (extended period), then extracted metabolites for evaluation of antibacterial and antibiofilm activities against clinical strains. GC–MS confirmed extract compositions. Results revealed these bacteria as promising sources of bioactive compounds, with metabolite activity and chemical profiles varying with fermentation period.

Extracts showed stronger activity against G+ve bacteria than G−ve strains (*E. coli* and *A. baumannii*). *E. coli* is resistant to all the extracts except E14, while *A. baumannii* is weakly sensitive to all the extracts. All the G+ve organisms were found to be sensitive to at least three of the four extracts. MRSA showed higher sensitivity to extracts, while the others only resisted one of the extracts obtained after 5 days (E5 and A5). The difference in sensitivity between G+ve and G−ve bacteria strains to the extracts may be due to their cell wall composition. G−ve resistance likely stems from their outer lipopolysaccharide membrane, which blocks hydrophobic compounds [39], unlike the thicker but permeable peptidoglycan layer in G+ve cells [24]. The outer membrane layer of G−ve bacteria is hydrophilic and filters hydrophobic molecules from penetrating the cell wall [40]. The 14‐day extracts outperformed their 5‐day counterparts. Extract E14 excelled, inhibiting all five tested strains (three G+, two G−), while E5 spared *S. aureus* ATCC 25923 and *E. coli*. Similarly, A14 targeted four strains versus A5’s three, with A5 uniquely inactive against *S. aureus*. Several factors such as fermentation period, media composition, and bacteria growth cycles are attributed to the metabolite production in bacteria [41]. The weak or moderate antibacterial activity observed, especially when treated with the extracts of a 5‐day fermentation period, may be due to the low antibiotic spectrum of the extract or the quantity not being enough to inhibit the bacterial cells [42]. This suggests an extended fermentation period may enrich inhibitory metabolites, targeting cell walls or membranes [43].

Three of the tested organisms were confirmed to form biofilm on surfaces, and the extracts were evaluated against them for antibiofilm activity, allowing the extracts to inhibit both planktonic and biofilm modes. They are *A. baumannii*. *S. aureus* ATCC 25923 and *S. aureus.* This dual action positions them as tools to combat biofilm‐related challenges in medical devices and infections. Results of the present study indicated a higher percentage (up to 84%) of biofilm inhibition by all the extracts against *S. aureus,* indicating the effectiveness of sponge‐associated bacterial metabolites as strong antibiofilm agents. Antibiofilm activity of the extracts also showed variations, with extracts of E5 (5‐day fermentation period) exhibiting higher activity than E14 against *S. aureus*. This difference may be due to the chemical composition of the extracts between two fermentation periods, as shown in supporting Tables 1 and 2. Prior studies confirm sponge‐associated bacteria yield antibiofilm metabolites against *S. aureus* and *A. baumannii* [44–47], acting via diverse mechanisms to prevent attachment and damage [48]. Here, all extracts showed activity; notably, *A. baumannii* is multidrug‐resistant [49], supporting the extracts’ role in addressing resistance. Among the bacteria strains used, *S. aureus* biofilms demand strategic control [50], and the metabolites of sponge‐associated bacteria offer novel options against chronic infections [51].

Fermentation duration critically influences production and activity of metabolites in sponge‐associated bacteria [52–54]. Prior works [22–24] have reported on the effect of fermentation period on the optimum production of metabolites from bacteria. Optimal periods for bioactive compound synthesis usually vary by strain, inoculum, nutrients, and pathways [55–57]. In this study, the dominance of fatty acid esters after 5 days of fermentation and diketopiperazines along with long‐chain alkanes after 14 days confirmed the role of the fermentation period in metabolite production. These changes in metabolite composition, particularly in extended incubation, may be due to stress responses to nutrient depletion and upregulating secondary metabolism [58], though overextension risks toxicity. When the fermentation period is longer than usual, the bacteria may produce new metabolites to respond to the stressful condition of nutrient depletion since metabolites are produced in response to changes in growth conditions [27]. While this may be required for some bacteria for optimum metabolite production, extending the fermentation period can have a negative effect on metabolite production because of the accumulation of toxic metabolites, which retard the growth of bacteria, or in some cases, lead to new metabolite production at a detectable level [21].

The antibacterial and antibiofilm activity differences observed in this study are linked to compositional shifts revealed by GC–MS in the extracts. Different metabolites will have different functions, and hence the bacteria will have varied activities [59] based on a variety of factors, including period of incubation, inoculum size, nutrient availability, environmental conditions, and the specific metabolic pathways involved [60, 61]. E14’s broad spectrum correlated with the presence of diketopiperazines (e.g., pyrrolo[1,2‐a]pyrazine‐1,4‐dione,hexahydro‐3‐(2‐methylpropyl), and aromatic acids), known for antimicrobial/antibiofilm effects against MRSA [17, 19, 20]. A14 shifted from fatty acid esters (A5) to long‐chain alkanes (e.g., eicosane, tetrapentacontane) and alcohols (e.g., n‐tridecan‐1‐ol), enhancing activity [58]. Eicosane (in both *Enterobacter* extracts) and indole also show antibacterial and antibiofilm action [55, 59]. n‐Tridecan‐1‐ol (observed in all extracts) is antibacterial, while tetrapentacontane (abundant in A14 and E5) is antimicrobial [20]. *Alcanivorax* extracts featured hexadecane and hexadecanoic acid ethyl ester, both reported as antibiofilm compounds [13–15]. While GC–MS spectra and heatmaps along with PCA analysis, indicated a metabolite shift with the fermentation period, library matching will provide only tentative identification. The PCA and heatmap of the compounds between different fermentation periods are provided as descriptive visualizations to explain the compositional differences. Hence, further structural characterization of these metabolites is required to understand the definitive composition shift in relation to fermentation period.

Overall, this study indicated the fermentation period‐driven changes in antibacterial and antibiofilm activities of metabolites produced by two sponge‐associated bacteria. The selective enrichment of some metabolites in the extracts obtained after 14 days of fermentation demonstrated the necessity of optimization of the fermentation period for increasing the bioactive potentials of bacteria associated with marine invertebrates. While GC–MS analysis indicated the shift in metabolite composition between two fermentation periods, the compound identification needs further studies. Future studies involving the purification and characterization of compounds using LC/MS and NMR may enhance their application in pharmaceutical and biotechnology sectors. This activity and compositional shifts between fermentation periods likely reflect metabolic pathway changes. Hence, genome mining will provide the details of biosynthetic pathways activated in different fermentation periods.

In conclusion, findings of this study demonstrated the influence of fermentation period on the bioactivity of two sponge‐associated bacterial strains. While strong antibacterial activity was observed in the extracts of a 14‐day fermentation period, short‐duration fermentation (5 days in this study) favors antibiofilm activity against *S. aureus*. The observed variations in metabolite compositions in GC–MS along with changes in antibacterial as well as antibiofilm activity in relation to the fermentation period highlight the importance of the fermentation period along with other factors for getting a higher yield of bioactive secondary metabolites from sponge‐associated bacteria.

## Author Contributions

Conceptualization, Sathianeson Satheesh; methodology, Sathianeson Satheesh; validation, Mamdoh T. Jamal, Sathianeson Satheesh; formal analysis, Sathianeson Satheesh; investigation, Mamdouh Al‐Harbi; resources, Mamdouh Al‐Harbi; data curation, Mamdoh T. Jamal; writing–original draft preparation, Mamdouh Al‐Harbi, Mamdoh T. Jamal; writing–review and editing, Sathianeson Satheesh; supervision, Mamdoh T. Jamal; project administration, Mamdouh Al‐Harbi; funding acquisition, Mamdouh Al‐Harbi.

## Funding

This project was funded by the Deanship of Scientific Research (DSR), King Abdulaziz University, Jeddah, under the Grant No. G:326‐150‐1442.

## Disclosure

All authors have read and agreed to the published version of the manuscript.

## Ethics Statement

The authors have nothing to report.

## Consent

The authors have nothing to report.

## Conflicts of Interest

The authors declare no conflicts of interest.

## Supporting Information

Additional supporting information can be found online in the Supporting Information section.

## Supporting information


**Supporting Information** Table S1. Composition of extract from *Enterobacter* sp. as revealed by GC–MS following 5 days of fermentation. Table S2. Composition of extract from *Enterobacter* sp. as revealed by GC–MS following 14 days of fermentation. Table S3. Composition of extract from *Alcanivorax* sp. as revealed by GC–MS following 5 days of fermentation. Table S4. Composition of extract from *Alcanivorax* sp. as revealed by GC–MS following 14 days of fermentation.

## Data Availability

The data that support the findings of this study are available from the corresponding author upon reasonable request.
