# Supplementary material for: Effects of Fermentation Period on Metabolite Production and Bioactivity in Two Sponge‐Associated Bacteria
Source: Scientifica (Cairo). 2026 Jul 29;2026:5979734. doi: 10.1155/sci5/5979734 (PMC13417491; doi:10.1155/sci5/5979734)
Supplement: Supplementary file 1 — Supporting Information Table S1. Composition of extract from Enterobacter sp. as revealed by GC–MS following 5 days of fermentation. Table S2. Composition of extract from Enterobacter sp. as revealed by GC–MS following 14 days of fermentation. Table S3. Composition of extract from Alcanivorax sp. as revealed by GC–MS following 5 days of fermentation. Table S4. Composition of extract from Alcanivorax sp. as revealed by GC–MS following 14 days of fermentation. [file SCI5-2026-5979734-s001.docx]

**Supplementary table**

**Effects of fermentation period on metabolite production and bioactivity in two sponge- associated bacteria**

Mamdouh Al-Harbi, Mamdoh T. Jamal, Sathianeson Satheesh*

Department of Marine Biology, Faculty of Marine Sciences, King Abdulaziz University,

Jeddah, Saudi Arabia*.*

*Corresponding Author, e-mail: [ssathianeson@kau.edu.sa](mailto:ssathianeson@kau.edu.sa)

Table S1. Composition of extract from *Enterobacter* sp. as revealed tentatively by GC-MS following 5 days fermentation.

|  |  |  |  |  |  |
| --- | --- | --- | --- | --- | --- |
| **Composition of extract from E5** | **Nature of compound** | **Retention time** | **Peak area** | **Range of activity** | **Reference** |
| Indole | Aromatic hydrocarbon | 6.062 | 13.2 | Antibiofilm/Antimicrobial | (Nieto et al. 2021) |
| n-Tridecan-1-ol | Long chain fatty alcohol | 7.041 | 3.87 | Antibacterial | (Togashi et al 2007) |
| Eicosane | Long chain alkane | 7.179 | 4.5 | Antibiofilm/Antimicrobial | (Ahsan et al 2017) |
| 1-Tetradecanol | Long chain fatty alcohol | 8.169 | 3.56 | Antibacterial | (Togashi et al 2007) |
| Hexadecanedioic acid, dimethyl ester | Fatty acid esters | 8.367 | 3.05 | _ |  |
| Pyrrolo[1,2-a]pyrazine-1,4-dione, hexahydro-3-(2-methylpropyl)- | Pyrollopyrazine | 9.383 | 2.64 | Antibiofilm/Antimicrobial | (Ser et al 2015; Kiran et al 2018; Rajivgandhi et al 2018). |
| Tetrapentacontane | Long chain alkane | 9.632 | 6.06 | Antibacterial | (Dhankhar et al 2013) |

Table S2. Composition of extract from *Enterobacter* sp. as revealed tentatively by GC-MS following 14 days fermentation.

| **Composition of extract from E14** | **Nature of compound** | **Retention time** | **Peak area** | **Range of activity** | **Reference** |
| --- | --- | --- | --- | --- | --- |
| Propanoic acid, 3-(methylthio)- | Carboxylic acid | 4.747 | 8.15 | _ |  |
| Benzeneacetic acid | Aromatic Fatty Acid | 5.816 | 15.6 | _ |  |
| n-Tridecan-1-ol | Long chain fatty alcohol | 7.042 | 3.31 | Antibacterial | (Togashi et al 2007) |
| Eicosane | Straight-chain alkane | 7.177 | 4.25 | Antibiofilm | (Ahsan et al 2017) |
| 3,6-Diisopropylpiperazin-2,5-dione | Piperazine | 8.981 | 5.06 | _ |  |
| l-Leucine, N-cyclopropylcarbonyl-, pentadecyl ester | Fatty acid esters | 9.325 | 4.7 | _ |  |
| Pyrrolo[1,2-a]pyrazine-1,4-dione, hexahydro-3-(2-methylpropyl)- | Pyrollopyrazine | 9.408 | 8.57 | Antibiofilm/Antimicrobial | (Ser et al 2015; Kiran et al 2018; Rajivgandhi et al 2018). |

Table S3. Composition of extract from *Alcanivorax* sp. as revealed tentatively by GC-MS following 5 days fermentation.

|  |  |  |  |  |  |
| --- | --- | --- | --- | --- | --- |
| **Composition of extract from A5** | **Nature of compound** | **Retention time** | **Peak area** | **Range of activity** | **Reference** |
| n-Tridecan-1-ol | Long chain fatty alcohol | 7.04 | 4.28 | Antibacterial | (Togashi et al 2007) |
| Cyclopropaneoctanoic acid, 2-octyl-, methyl ester, cis- | Fatty acid esters | 7.823 | 13.3 | _ |  |
| 3-Chloropropionic acid, heptadecyl ester | Fatty esters | 8.167 | 2.17 | _ |  |
| Hept-2-ene, 2,4,4,6-tetramethyl- | Branched chain Alkene | 8.349 | 9.16 | _ |  |
| l-(+)-Ascorbic acid 2,6-dihexadecanoate | Fatty esters | 9.422 | 7.25 | _ |  |
| Hexadecanoic acid, ethyl ester | Fatty esters | 9.571 | 2.13 | Antibiofilm | (Dusane et al 2011 Shaaban et al 2021) |

Table S4. Composition of extract from *Alcanivorax* sp. as revealed tentatively by GC-MS following 14 days fermentation

| **Composition of extract from A14** | **Nature of compound** | **Retention time** | **Peak area** | **Range of activity** | **Reference** |
| --- | --- | --- | --- | --- | --- |
| n-Tridecan-1-ol | Long chain fatty alcohol | 7.041 | 5.43 | Antibacterial | (Togashi et al 2007) |
| Hexadecane | Long chain Alkane | 7.417 | 2.48 | Antibiofilm | (Sharma and Mallubhotla 2022) |
| Allyldimethyl(prop-1-ynyl)silane | Organosilicon | 8.348 | 12.4 | _ |  |
| l-(+)-Ascorbic acid 2,6-dihexadecanoate | Fatty esters | 9.421 | 1.65 | _ |  |
| Tetrapentacontane | Long chain Alkane | 9.746 | 17.5 | Antibacterial | (Dhankhar et al 2013) |

References

Ahsan T, Zang C, Yu S et al (2022) Screening, and Optimization of Fermentation Medium to Produce Secondary Metabolites from Bacillus amyloliquefaciens, for the Biocontrol of Early Leaf Spot Disease, and Growth Promoting Effects on Peanut (Arachis hypogaea L.). J Fungi 8:1223. <https://doi.org/10.3390/jof8111223>

Dhankhar S, Dhankhar S, P. Yadav J. Investigating Antimicrobial Properties of Endophytic fungi Associated with Salvadora oleoides Decne. Anti-Infective Agents. 2013;11(1):49-59. <https://doi.org/10.2174/22113626130106>

Dusane DH, Pawar VS, Nancharaiah YV, Venugopalan VP, Kumar AR, Zinjarde SS. Anti-biofilm potential of a glycolipid surfactant produced by a tropical marine strain of Serratia marcescens. Biofouling. 2011;27(6):645-654. <https://doi.org/10.1080/08927014.2011.594883>.

Kiran GS, Priyadharsini S, Sajayan A, Ravindran A, Selvin J. An antibiotic agent pyrrolo[1,2-a]pyrazine-1,4-dione,hexahydro isolated from a marine bacteria Bacillus tequilensis MSI45 effectively controls multi-drug resistant Staphylococcus aureus. RSC Advances. 2018;8(32):17837-17846. <https://doi.org/10.1039/C8RA00820E>

Nieto MJ, Lupton HK. Indole and Indoline Scaffolds in Antimicrobials: Overview, Synthesis and Recent Advances in Antimicrobial Research. Current Medicinal Chemistry. 2021;28(24):4828-4844. https://doi.org/10.2174/0929867327666201102114923.

Rajivgandhi G, Vijayan R, Maruthupandy M, Vaseeharan B, Manoharan N. Antibiofilm effect of Nocardiopsis sp. GRG 1 (KT235640) compound against biofilm forming Gram negative bacteria on UTIs. Microbial Pathogenesis. 2018;118:190-198. <https://doi.org/https://doi.org/10.1016/j.micpath.2018.03.011>.

Ser H-L, Palanisamy UD, Yin W-F, Abd Malek SN, Chan K-G, Goh B-H, Lee L-H. (2015) Presence of antioxidative agent, Pyrrolo[1,2-a]pyrazine-1,4-dione, hexahydro- in newly isolated Streptomyces mangrovisoli sp. nov. Frontiers in Microbiology. 6(854). <https://doi.org/10.3389/fmicb.2015.00854>

Shaaban MT, Ghaly MF, Fahmi SM. (2021) Antibacterial activities of hexadecanoic acid methyl ester and green-synthesized silver nanoparticles against multidrug-resistant bacteria. J Basic Microbiol. 61(6):557-568. <https://doi.org/10.1002/jobm.202100061>.

Togashi N, Shiraishi A, Nishizaka M, Matsuoka K, Endo K, Hamashima H, Inoue Y. (2007) Antibacterial activity of long-chain fatty alcohols against Staphylococcus aureus. Molecules.12(2):139-48. <https://doi.org/10.3390/12020139>
